# The impact of pharmacist intervention on prophylactic antibiotics use in orthopedic surgery at a hospital in China

**DOI:** 10.1097/MD.0000000000028458

**Published:** 2021-12-30

**Authors:** Hong Zhou, Lihong Liu, Xiao Sun, Huaguang Wang, Xiaojia Yu, Ye Su, Zhaoyuan You, Zhuoling An

**Affiliations:** a Department of Pharmacy, Beijing Chaoyang Hospital, Capital Medical University, Beijing, People's Republic of China; b Department of Pharmacy, China-Japan Friendship Hospital, Beijing, People's Republic of China; c Department of Epidemiology, School of Public Health, Tulane University, LA; d Nursing Department, Beijing Chaoyang Hospital, Capital Medical University, Beijing, People's Republic of China.

**Keywords:** anti-bacterial agents, antibiotic prophylaxis, antimicrobial stewardship, orthopedics

## Abstract

This study aimed to assess the impact of the pharmacist-led intervention on perioperative antibiotic prophylaxis by standardizing the cephalosporin intradermal skin test in the orthopedic department.

A pre-and postintervention study was conducted among patients in the Orthopedics Department at the Beijing Chao-Yang Hospital in China. Use of intradermal skin test, perioperative antibacterial prophylaxis, and cost of care were compared between the preintervention population (admitted from 6/1/2018 to 8/31/2018) and postintervention population (admitted from 1/1/2019 to 3/31/2019). Logistic regression and generalized linear regression were used to assess the intervention impact.

425 patients from the preintervention period and 448 patients from the postintervention period were included in the study. After the implementation of the pharmacist intervention program, there was a decrease in the utilization of intradermal skin tests, from 95.8% to 16.5% (*P* < .001). Patients were more likely to have cephalosporin as prophylactic antimicrobials (OR = 5.28, *P* < .001) after the implementation. The cost of antimicrobials was significantly reduced by $150.21 (*P* < .001) for each patient.

Pharmacist-involved intervention can reduce the utilization of cephalosporins skin tests and decrease the prescription of unnecessary high-cost antimicrobials.

## 1 Introduction

Cephalosporins are currently one of the most commonly prescribed antimicrobials due to their broad spectrum of antimicrobial activity, low toxicity profile, and good pharmacoeconomic properties worldwide.^[1]^ According to global guidelines, cephalosporins are recommended for the prevention of surgical site infections as the first-line prophylaxis for a variety of surgical procedures.^[2]^

Nevertheless, beta-lactam antimicrobials including penicillin and cephalosporins are the most common class of drugs suspected when drug hypersensitivity reactions occur.^[3]^ The prevalence of beta-lactam antimicrobials allergy goes from 0.7% to 10% in the general population.^[4]^ Anaphylactic and anaphylactoid reactions occurring during anesthesia are also concerns of medical practitioners since they are usually unpredictable and may be potentially life-threatening even when appropriately treated. Several cases of perioperative anaphylaxis caused by cephalosporins have been reported.[^5^,^6^] In a 2 years survey of such reactions in France, penicillin and cephalosporins were identified as the offending agents in 33 and 31 cases, respectively, from 518 cases of perioperative anaphylaxis.^[7]^

However, overdiagnosis of beta-lactam allergy is a significant and growing public health problem. Once an individual is considered allergic to beta-lactam antimicrobials, it was rarely questioned, re-evaluated, or verified. Even worse, due to lack of knowledge about beta-lactam allergy, some physicians developed a prescribing habit to cross off all beta-lactam antimicrobials for patients with a history of allergy.[^8^,^9^,^10^] Moreover, in China, in order to avoid allergic reactions during perioperative, to reduce the medical risks or conflicts between doctors and patients, the intradermal skin tests of cephalosporins were conducted on all patients regardless of drug allergic history and the false-positive rate was high.[^11^,^12^]

For years, pharmaceutical care has been a key strategy to improve healthcare safety. Pharmaceutical care is a collaborative care approach which implies all the actors of the medication circuit in order to prevent and correct drug-related problems that can lead to adverse drug events.^[13]^ One way to improve medication safety in hospitals is to integrate clinical pharmacists into the medication process. According to available data, the integration of a clinical pharmacist in multi-professional teams during admission, hospitalization and discharge can significantly reduce drug-related problem, costs and increases efficacy of drug therapy.^[14]^ In terms of antimicrobial stewardship, pharmacists are acknowledged as health care professionals specializing in pharmacotherapy outcomes and management, responsibility is to conserve the effectiveness of antimicrobials and to promote appropriate antibiotic use.^[15]^

Fluoroquinolones, clindamycin, vancomycin, and other broad-spectrum antimicrobials as alternatives have led to worse clinical outcomes, increased incidence of serious antibiotic-resistant infections, and prolonged hospitalizations.^[16]^ In 2016, the Infectious Diseases Society of America antimicrobial stewardship guidelines emphasized the burden of antibiotic allergy and recommended that the assessment of antibiotic allergy should be included in antimicrobial stewardship programs.^[17]^ This study aimed to assess the impact of the pharmacist intervention on perioperative antibiotic prophylaxis by standardizing the cephalosporin intradermal skin test in orthopedic department.

## 2 Methods

### 2.1 Settings and study population

The study was a pre-post intervention study conducted in the Orthopedics Department at the Beijing Chao-Yang Hospital, Capital Medical University. Changes in perioperative antibiotic prophylaxis were compared between 425 patients from the preintervention period (from June 1, 2018, to August 31, 2018) and 448 patients from the postintervention period (from January 1, 2019, to March 31, 2019).

All patients admitted to the hospital for the following orthopedic procedures were included in this study: repair and plastic operations on joint structures, reduction of fracture and dislocation, operations on muscle, tendon, fascia, and bursa, incision and excision of joint structures, operations on the spinal cord and spinal canal structures, and other operation on bones except facial bones. Patients were excluded if they had any of the listed conditions:

1. patients had no surgical indications after evaluation;
2. only clean orthopedic procedures were needed without the use of prophylaxis;
3. patients had a diagnosis of infectious disease such as staphylocoelitis;
4. patients had preoperative infections and were using antimicrobials;
5. patients had grade III open fractures with extensive soft tissue damage and crushing;
6. patients were transferred to the surgical intensive care unit after operations.

### 2.2 Interventions

Pharmacists who participated in antimicrobial stewardship projects for perioperative prophylaxis developed the following interventions:

1. joining the treatment team and participating in ward rounds;
2. conducting medication reconciliation;
3. inquiring and reassessing the patient's allergy history;
4. providing a standard concentration of skin test solution by the pharmacy intravenous admixture services with the concentration of 300 μg/mL of the original drug;
5. providing training to all the medical staff of the orthopedics department on limiting intradermal skin test only to the patients with allergic history using the standard concentration solution provided by pharmacy intravenous admixture services.

**Figure 1:**
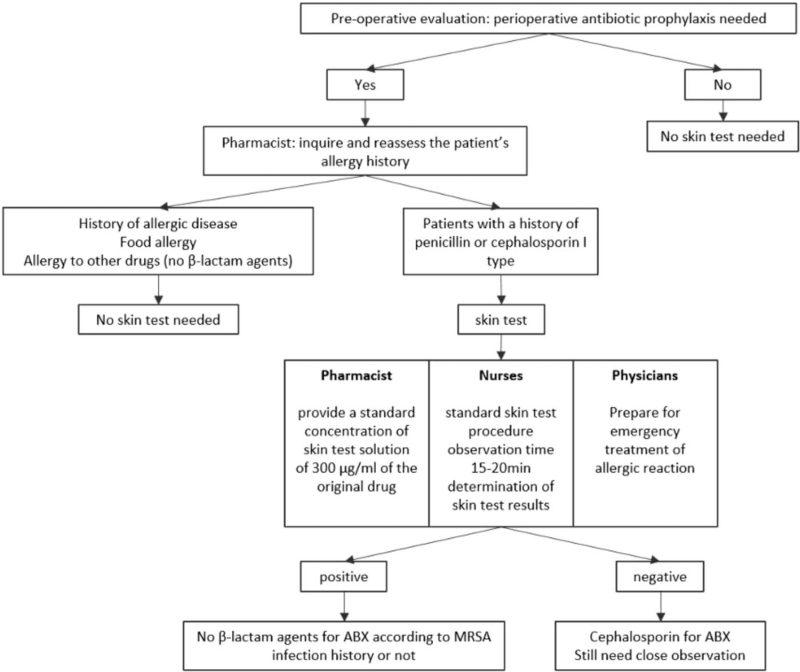
Intervention Flow Chart.

The detailed intervention process flow is presented in Figure 1.

### 2.3 Data source

Patients’ demographic information, diagnosis, clinical outcomes, and antimicrobial data were manually retrieved from the electronic health record system of the hospital. Surgical procedures were recorded using the International Classification of Diseases, 9th version, Procedure Codes (ICD -9-PCS).

The following data were collected:

1. demographics like patient gender and age;
2. admission and discharge diagnosis and case mix index (CMI) which indicates patients’ condition severity;
3. procedure type according to the ICD-9-PCS and duration of surgery;
4. allergy information including food, drug, and disease allergic history, the results of intradermal skin tests, and the allergic reactions after medication;
5. perioperative antibiotic prophylaxis data such as antimicrobial agents, duration and adverse effects;
6. pharmacoeconomic indicators such as total drug cost and antibiotic cost.

The protocol was approved by the Drug and Therapeutics Committee and the Ethics Committee of Beijing Chao-Yang Hospital, Capital Medical University (Approval number: 2017-11-28-3).

### 2.4 Data analysis

The data were analyzed using SPSS software (version 24.0; SPSS, Inc, Chicago, IL). The Mann–Whitney tests were conducted for the numerical measures with non-normal distributions. Pearson Chi-Squared tests were used for categorical variables. *P* < .05 was considered to indicate statistical significance.

## 3 Results

During the pre-and post-intervention periods, a total of 1611 patients were admitted to the Orthopedic Department. Of 784 patients in the pre-intervention period, 21 were excluded for not having surgical indications, 230 for undergoing clean orthopedic procedures without the use of prophylaxis, 8 for having infectious diseases, 39 for having preoperative infections and using antimicrobials, 6 for having grade III open fractures with extensive soft tissue damage and crushing, 55 for being admitted to the surgical intensive care unit after the operation. In the postintervention period, 9 out of 827 patients were excluded for not having surgical indications, 258 for undergoing clean orthopedic procedures without the use of prophylaxis, 3 for having infectious diseases, 54 for having preoperative infections and using antimicrobials, 3 for having Grade III open fractures with extensive soft tissue damage and crushing, and 52 for being admitted to the surgical intensive care unit. In the end, a total of 873 patients were included in this study, of which 425 were from the pre-intervention period and 448 were from the postintervention period.

There was no significant difference between pre-and postintervention populations in gender, length of stay, and CMI. Postintervention population is slightly older than the preintervention population. The demographic and procedural characteristics of the 2 populations are shown in Table 1.

**Table 1:** Demographic and procedural data by pre- and postintervention groups.

Characteristics	Preintervention (n = 425)	Postintervention (n = 448)	*P*
Female, frequency (%)	224 (57.4%)	239 (53.3%)	.247
Age, mean (SE)	52.4 (0.897)	58.0 (0.824)	.047
Type of procedure, frequency (%)			<.001
* Repair and plastic operations on joint structures*	159 (37.4%)	184 (41.1%)	
* Other operation on bones, except facial bones*	105 (24.7%)	91 (20.3%)	
* Reduction of fracture and dislocation*	103 (24.2%)	97 (21.7%)	
* Operations on muscle, tendon, fascia, and bursa, except hand*	27 (6.4%)	16 (3.6%)	
* Incision and excision of joint structures*	22 (5.2%)	12 (2.7%)	
* Operations on spinal cord and spinal canal structures*	9 (2.1%)	41 (9.2%)	
* Others*	0 (0%)	7 (1.6%)	
Length of stay	11.47 (0.332)	11.01 (0.283)	.293
CMI, mean (SE)	1.50 (0.034)	1.55 (0.033)	.317

In Table 2, patients’ allergic information was compared between the 2 populations. Asthma is the most common history allergy which represents 1.6% and 0.4% in pre and postintervention groups. The top 3 most common previous drug allergy history were penicillins (25, 5.9% in preintervention; 17, 3.8% in postintervention), sulfonamides (10, 2.4% in preintervention; 15, 3.3% in postintervention) and cephalosporins (3, 0.7% in preintervention; 5, 1.1% in postintervention).

**Table 2:** Allergic data pre-and postintervention.

	Preintervention (n = 425)	Postintervention (n = 448)
Previous allergic disease history		
Asthma	7 (1.6%)	2 (0.4%)
Allergic rhinitis	0 (0.0%)	2 (0.4%)
Urticaria	1 (0.2%)	1 (0.2%)
Anaphylactoid purpura	0 (0.0%)	1 (0.2%)
Food allergy	1 (0.2%)	1 (0.2%)
Previous drug allergic history		
Penicillins	25 (5.9%)	17 (3.8%)
Sulfonamides	10 (2.4%)	15 (3.3%)
Cephalosporins	3 (0.7%)	5 (1.1%)
Tetanus antitoxin	2 (0.5%)	2 (0.4%)
Chinese patent medicine	2 (0.5%)	0 (0.0%)
SMZCo	1 (0.2%)	0 (0.0%)
Macrolides	1 (0.2%)	1 (0.2%)
Furazolidone	0 (0.0%)	1 (0.2%)
Metoclopramide	1 (0.2%)	1 (0.2%)
Ephedrine	1 (0.2%)	0 (0.0%)
Nitroglycerin	1 (0.2%)	0 (0.0%)
Iodine	1 (0.2%)	0 (0.0%)
Alcohol	0 (0.0%)	2 (0.4%)

The number of patients receiving intradermal skin tests decreased from 95.8% (407) in pre- to 16.5% (74) in postintervention (*P* < .001). In the postintervention period, there was an increase in the use of cephalosporin for perioperative antibacterial prophylaxis from 83.5% (355) to 96.6% (433) (*P* < .001). On the contrary, there was a decrease in the use of suboptimal antimicrobials for prophylaxis, such as vancomycin from 8.5% (36) to 1.6% (7) (*P* < .001), clindamycin from 8.5% (36) to 1.6% (7) (*P* < .001), etc. From the cost perspective, although there was no significant difference in the total drug expenditure per patient (stands for the cost of all therapeutic drugs used during each patient's hospitalization, including antibacterial drugs, analgesics, anticoagulants, and treatment drugs for chronic diseases [if the patient has underlying chronic diseases]) (*P* = .211) between the 2 groups, the average cost of antimicrobials decreased from $49.7 to $44.2 (*P* = .024) with antimicrobials cost proportion dropped from 16.8% to 13.8% (*P* < .001). Findings of utilization and cost of intradermal skin tests and perioperative antibacterial prophylaxis were summarized in Table 3.

**Table 3:** Intradermal skin test, perioperative antibacterial prophylaxis, and cost data pre-and postintervention.

	Preintervention (n = 425)	Postintervention (n = 448)	*P*
Patients with intradermal skin test	407 (95.8%)	74 (16.5%)	<.001
POABP			<.001
Cephalosporin	355 (83.5%)	433 (96.6%)	<.001
Vancomycin	36 (8.5%)	7 (1.6%)	<.001
Clindamycin	28 (6.6%)	4 (0.9%)	<.001
Levofloxacin	6 (1.4%)	4 (0.9%)	.537
Cost			
Total drugs per patient ($)	349.2 (191.5, 577.8)	375.9 (237.6, 567.8)	.211
Antimicrobials per patient ($)	49.7 (32.1, 82.8)	44.2 (28.7, 71.6)	.024
Antibiotic cost percentage	16.8% (11.4%, 25.1%)	13.8% (8.6%, 21.4%)	<.001

Table 4 presents the results from logistic regressions estimating the likelihood of patients getting intradermal skin test and the usage of prophylactic antimicrobials, controlling for patients’ age, gender, length of inpatient stay, and CMI. Compared to preintervention population, postintervention population was significantly less likely to undergo an intradermal skin test (OR: 0.008, 95% CI: 0.005–0.014). Patients in postintervention group had a 5.28-fold higher likelihood (95% CI: 2.95–9.43) of having cephalosporin as prophylactic antimicrobials compared with who were in preintervention group. We also examined the impact of the intervention on the total drug cost and the cost of antimicrobials, adjusted for patients’ age, gender, length of inpatient stay, and CMI. There was no difference in the total drug cost between pre- and postintervention groups. On average, patients in the postintervention group had lower antimicrobials cost and lower antimicrobials cost percentage than patients in the preintervention group, by $150.21 (95% CI: −225.00–−75.43) and 3.2% (95% CI: −4.7%–−1.7%), respectively.

**Table 4:** Impact of the intervention on intradermal skin test, prophylactic antibiotics, and drug costs.

	Effect size^∗^ (95% confidence intervals)	*P*
Patients with intradermal skin tests, OR	0.008 (0.005, 0.014)	<.001
Cephalosporin as POABP, OR	5.28 (2.95, 9.43)	<.001
Cost		
Total drugs per patient ($), Coef	–7.34 (–165.61, 150.93)	.928
Antibiotics cost per patient ($), Coef	–150.21 (–225.00, –75.43)	<.001
Antibiotics cost percentage, Coef	–3.2% (–4.7%, –1.7%)	<.001

## 4 Discussion

At the G20 in Hangzhou China in 2017, antimicrobial resistance has been recognized as one of the top 5 factors impacting the world economy. China has carried out a 3-year special rectification activity on the clinical application of antimicrobials nationwide since 2011. In 2016, China launched the national action plan to Contain Antimicrobial Resistance (2016–2020). Several studies have demonstrated that the pharmacists’ participation could promote the reasonable use of antimicrobials both in preventive and therapeutic applications.[^18^,^19^,^20^] Especially in the perioperative prophylactic application of antimicrobials, the perioperative-dose timing, the selection, the dosing, and the duration of prophylaxis have been greatly improved. However, we are faced with another problem of over-utilization of intradermal skin tests for penicillin and cephalosporins in inpatient settings.

In 2012, penicillin and cephalosporin were the top 2 antimicrobials sold in China, making up nearly 70% of antibacterial drug market.^[21]^ However, the annual report of national adverse drug reaction monitoring of China showed that a total of 1.4 million adverse drug reaction events were identified in 2017, among which about 508 thousand cases were related to antimicrobial agents, with cephalosporins ranked the first. The top 3 systems and organs affected by adverse drug reactions were skin and appendages disorders (24.2%), body as whole-general disorders (18.6%), and resistance mechanism disorders (11.4%). Unfortunately, a lot of physicians still lack knowledge on the safe use of β-lactams.^[22]^ Because there are no clear regulations in the Chinese pharmacopeia and the medicine specification, 3 types of situations could happen in practice, administering without intradermal skin test, substituting of cefazolin or penicillin intradermal skin test, and ordering drug intradermal skin test.^[11]^ In our hospital, the physicians are accustomed to conducting routine original drug intradermal skin tests before using cephalosporins to avoid allergy. However, in some circumstances, due to false-positive outcomes of the intradermal skin test, physicians could not use first-line antimicrobials and had to choose suboptimal alternatives, which led to increased treatment costs and increased risk of antibiotic resistance.

The penicillin skin tests have been proved for the diagnosis and indications of IgE-mediated hypersensitivity to penicillin.[^23^,^24^] Whereas, although cephalosporin intradermal skin tests are widely used in China, Korea, and other Asian countries,^[25]^ there is still much controversy. Some researchers claimed it could accurately predict immediate hypersensitivity.^[26]^ But some scholars had the opposite opinions, S.-Y. Yoon developed a prospective study by conducting intradermal skin tests with 4 different generations of cephalosporins. 74 (5.2%) out of 1421 patients were positive to at least 1 cephalosporin but none of the responders had immediate hypersensitivity reactions after a challenge dose of the same or different cephalosporin. Therefore, inferred the positive predictive value is 0%.^[27]^

Our study is one of the earliest study to examine the impact of eliminating unnecessary penicillin skin-test through an pharmacists-participated intervention in the antibiotic stewardship program. In our study, pharmacists participated in the clinical practice, evaluated patients’ allergic history, and recommended that intradermal skin tests should not be performed on patients without a drug allergic history. Patients with allergic history should conduct the intradermal skin test of the original drug with a standard 300 μg/mL concentration solution which was dissolved at pharmacy intravenous admixture service. The intervention avoided unnecessary intradermal skin tests, substantially reduced the prescription of suboptimal antimicrobials, and significantly lowered the antimicrobial expenditure.

Although our study showed promising results in population with orthopedics surgery, we recognized that our results are not representative of the general inpatient population, and we also acknowledge that our approach may not be applicable to patients with non-elective surgeries during emergent situations. Another outcome that we were not able to evaluate is the false positive results of the patients with positive skin tests. Because those patients did not go through cephalosporins therapy. Further research will be needed to assess the false-positive rate and innovative antibiotic stewardship intervention is needed to identify and un-label false-positive cases.

## 5 Conclusion

Pharmacist involved intervention can reduce the utilization of cephalosporins skin tests and decrease the prescription of unnecessary higher cost antimicrobials such as vancomycin, clindamycin, and levofloxacin as the perioperative prophylaxis.

## Author contributions

**Formal analysis:** Xiao Sun.

**Investigation:** Xiaojia Yu, Ye Su, Zhaoyuan You.

**Supervision:** Lihong Liu.

**Validation:** Huaguang Wang.

**Writing – original draft:** Hong Zhou.

**Writing – review & editing:** Zhuoling An.
